# Rescue Thalamotomy for Habituation to Deep Brain Stimulation in Essential Tremor: Case Report

**DOI:** 10.5334/tohm.1050

**Published:** 2026-02-09

**Authors:** Roman Kiselev, Vladislav Babchenko

**Affiliations:** 1 Meshalkin National Medical Research Centre, Novosibirsk, Russian Federation

**Keywords:** dbs, tremor, thalamotomy, habituation

## Abstract

**Background:**

Habituation to deep brain stimulation (DBS) remains a therapeutic challenge in essential tremor (ET), with 4–42% of patients experiencing progressive loss of benefit.

**Case report:**

A 68-year-old man with ET presented with debilitating bilateral tremor. The patient underwent bilateral DBS to posterior subthalamic area (PSA). Despite initial tremor relief, symptoms gradually recurred three months later. After 12 months of unsuccessful programming, the patient underwent left-sided radiofrequency Vim thalamotomy. Due to severe tremor rebound phenomenon, tremor control was achieved in 1.5 months.

**Discussion:**

To our knowledge, this study represents the first report of successful thalamotomy performed for PSA-DBS habituation, accompanied by delayed postoperative tremor improvement. The clinical trajectory and putative mechanisms underlying both habituation and lesion-induced tremor suppression are discussed based on imaging analysis.

## Introduction

Deep brain stimulation (DBS) of the posterior subthalamic area (PSA) is an effective treatment for pharmacologically resistant essential tremor (ET). However, some patients experience a gradual decline in therapeutic efficacy over time. Reported rates of diminished benefit range from 4% to 42% [1], with this phenomenon primarily observed in DBS targeting the ventrointermediate nucleus (Vim) and rarely in PSA stimulation.

The management of habituation remains a significant challenge for clinicians. Ablative surgery may represent a potential option in selected cases. The few available studies have reported mixed outcomes following rescue Vim thalamotomy, ranging from no functional improvement [2] to complete tremor resolution [3]. The present report highlights Vim thalamotomy as a viable therapeutic option for patients with PSA-DBS habituation and emphasized the clinical course characterized by delayed postoperative improvement.

Tremor severity was assessed using the Essential Tremor Rating Assessment Scale (TETRAS performance score), and its impact on daily living was quantified via the TETRAS activities of daily living (ADL) subscale. This paper adheres to the CARE guidelines for case reporting [4].

## Case report

A 68-year-old man with 50-year ET history presented with debilitating bilateral tremor. Over the three years prior to admission, tremor progression reached a severity that profoundly impaired his daily activities (TETRAS ADL score: 30). Neurological examination revealed isolated action tremor in arms (TETRAS performance score: 30.5) without other abnormalities. A diagnosis of essential tremor was confirmed based on the 2018 International Parkinson and Movement Disorder Society (IPMDS) consensus criteria [5].

**Figure 1:**
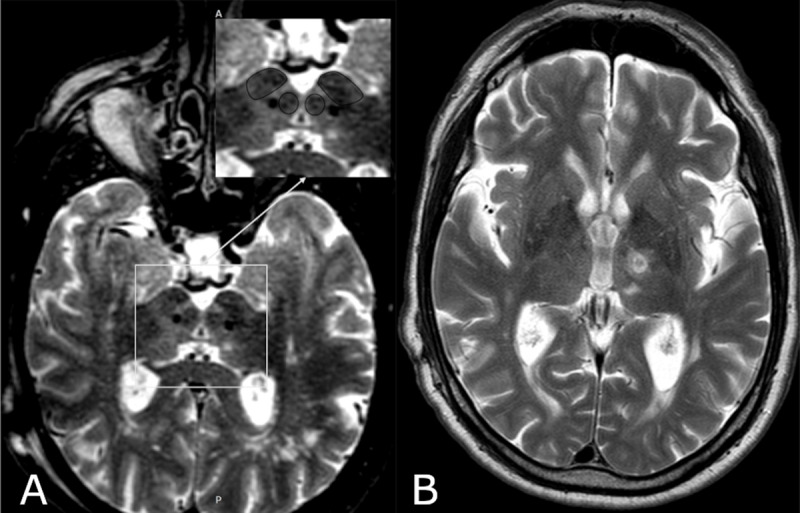
**(a)** Postoperative T2-weighted axial MRI demonstrating bilateral DBS lead placement in the posterior subthalamic area. The enlarged inset delineates anatomical boundaries of the subthalamic nucleus (STN) and red nucleus (RN). **(b)** Corresponding T2-weighted image showing the radiofrequency lesion in the left ventrolateral thalamus (Vim).

Since 2018, PSA has been the preferred target for ET at the Meshalkin National Medical Research Centre, based on published evidence [6,7,8,9] and institutional experience. Trajectories are planned through Vim and verified at the level of AC-PC level to optimize efficacy. In our experience, ventral contacts within PSA most often provide the best balance between tremor control and side effects. Bilateral implantation of DBS leads (model 3389, Medtronic) into the PSA was performed using a stereotactic CRW frame (Integra). The target was localized just lateral to the medial border of the subthalamic nucleus (STN), along the midline axis of the red nuclei at the level of their maximum diameter. Intraoperative macrostimulation demonstrated significant tremor relief with low total electrical energy delivered (TEED). Postoperative MRI confirmed accurate lead placement (Figure 1A). Initial programming on the seventh postoperative day achieved significant tremor reduction at low stimulation parameters (left lead: 60 µs, 130 Hz, 0.9 mA; right lead: 60 µs, 130 Hz, 1.1 mA).

However, symptoms gradually recurred three months later. Temporary improvement was observed with parameter adjustments, but subsequent reprogramming sessions — including amplitude and frequency increases, bipolar stimulation, and interleaved mode — yielded only transient benefits. Further TEED escalation (left lead: 60 µs, 180 Hz, >2.7 mA; right lead: 60 µs, 180 Hz, >3.2 mA) induced side effects (dysarthria, gait ataxia). When stimulation was temporarily discontinued (“stimulation holiday”), we observed a rebound phenomenon with tremor amplitude exceeding preoperative levels, ultimately worsening the patient’s functional status (TETRAS performance score: 34; ADL score: 32).

After 12 months of unsuccessful programming, the patient underwent DBS system explantation and left-sided radiofrequency thalamotomy through the existing burr hole. Due to the higher risk of hemorrhagic complications [10,11], microelectrode recording was not routinely employed. To verify the electrode location and assess stimulation efficacy and potential side effects, intraoperative macrostimulation (100 µs, 100 Hz, up to 4.0 mA) was performed, followed by a test ablation at 50°C for 30 seconds. Along the trajectory, two thermocoagulations of the Vim nucleus were applied using a monopolar radiofrequency probe (tip diameter = 1.6 mm; length = 3 mm) at 75°C for 60 seconds (Figure 1B).

The thalamotomy procedure was completed without perioperative complications or adverse effects. However, only slight tremor relief was achieved initially. We observed a delayed treatment response characterized by gradual tremor resolution in the contralateral arm (TETRAS: performance score = 21.5, ADL score = 17) and ipsilateral tremor reduction to pre-DBS baseline level over 1.5 months. Clinical efficacy persisted for 10 months postoperatively (Supplementary Video). Despite an ischemic stroke (left-sided hemiparesis) occurring at 14-month follow-up, thalamotomy-mediated tremor control remained effective on the right side.

**Supplementary Video:** Severity of patient’s tremor before leads implantation, during intraoperative macrostimulation, after initial programming, after 12 months of unsuccessful stimulation adjustments and 10 months after thalamotomy.

## Discussion

The pathophysiology of essential tremor (ET) is thought to involve cerebellar neurodegeneration and dysfunction [12]. These pathological changes produce tremor-related oscillations within the cerebellothalamocortical (CTC) circuit (the “oscillating network hypothesis”).

The therapeutic effects of DBS are both site-specific and frequency-dependent [13]. As shown by Ranck (1975), electrical stimulation preferentially targets axons rather than cell bodies due to their lower activation thresholds [14]. Low-frequency stimulation (below the synaptic depression threshold) enhances neural output in excitatory CTC fibers, while high-frequency stimulation initially induces an excitatory response that weakens over time [15].

Recent evidence suggests that habituation may occur when the distance between the DBS lead and the cerebellothalamocortical tract (CTT) is suboptimal [8,16]. This aligns with the identified “sweet spot” for effective tremor control in essential tremor, as demonstrated in a recent multicenter study [17], as it located at the convergence of decussating and non-decussating fibers of the CTT.

**Figure 2:**
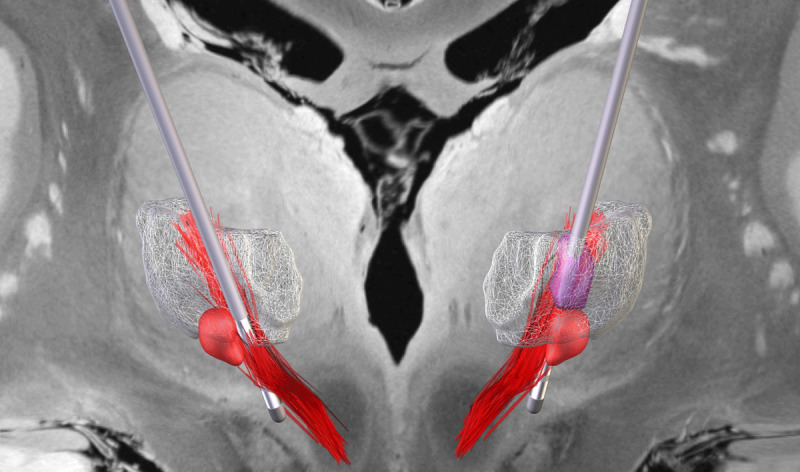
3D reconstruction of DBS leads (silver) and thalamotomy lesion (necrotic core (purple) and perilesional edema (pink)) registered to Essential Tremor Probabilistic Mapping atlas in MNI space. Lead trajectories intersect the decussating fibers of the cerebellothalamic tract (CTT, red) bilaterally, while the lesion core localizes in the Vim of the left thalamus (white transparent). (Coordinate space: MNI152; visualization software: Lead-DBS v3.2).

To evaluate leads positioning in our case, we performed lead localization and reconstruction using the Lead-DBS toolbox (v3.2) in MATLAB R2024a (The MathWorks, Natick, MA). Postoperative computed tomography (CT) scans were linearly coregistered to the preoperative MRI images (T1- and T2-weighted) using Advanced Normalization Tools (ANTs). ANTs was subsequently employed to normalize images to the ICBM 2009b MNI space. DBS leads were automatically reconstructed using PaCER method, and the resulting reconstructions were overlaid onto the template space of the Essential Tremor Probabilistic Mapping atlas [17]. The Vim was visualized at a probability threshold of 0.5. The reconstruction confirmed bilateral decussating CTT coverage (Figure 2), explaining high initial efficacy and ruling out malposition. However, unaddressed non-decussating fibers may permit habituation via glutamatergic potentiation. We propose that DBS habituation arises from incomplete CTT circuit inhibition, leading to progressive antidromic potentiation of excitatory glutamatergic activity under chronic stimulation. Habituation has been observed with Vim and PSA stimulation but not with STN or GPi targets. This difference may reflect the predominance of glutamatergic excitatory inputs in Vim and PSA, in contrast to the mainly GABAergic GPi and more balanced distribution of the STN. A link between cerebellar signs and habituation has also been noted: a PET study showed increased cerebellar glucose uptake during stimulation, possibly due to antidromic CTT activation [18]. Our patient exhibited marked tremor rebound, while gait ataxia appeared only at higher stimulation intensities. However, despite the absence of subjective complaints, no formal objective assessment of ataxia was performed. The mechanism of antidromic potentiation may explain the transient improvement with TEED escalation (via an enlarged volume of tissue activated, VTA), ultimately limited by stimulation-induced side effects (e.g., dysarthria, ataxia) at higher intensities; and the “tremor rebound” phenomenon observed after DBS discontinuation, where the absence of modulation unmasks accumulated pathological activity, exacerbating tremor beyond preoperative severity.

A key question remains: Why did thalamotomy succeed when DBS failed? The therapeutic mechanisms differ fundamentally between these interventions. While DBS effects depend on selective, frequency-dependent modulation of neural elements, thalamotomy’s benefits derive from definitive lesion characteristics (volume and location) that indiscriminately interrupt pathological circuitry. Also, key distinctions emerge from anatomical targeting: subthalamic DBS primarily modulates cerebello-thalamic fibers, while thalamic intervention affects both cerebello-thalamic and thalamocortical projections [19]. Our findings support the “thalamic filter hypothesis”, wherein complete thalamic neuronal inhibition appears necessary for sustained tremor control [15,17]. This inhibition is necessary to decouple pathological thalamocortical from corticospinal reflex loops.

The delayed tremor relief (1.5-month post-thalamotomy) warrants investigation. Intraoperative macrostimulation produced only mild tremor improvement, probably related to the rebound phenomenon. Although no adverse events or complications occurred, early post-thalamotomy tremor control remained unsatisfactory. Postoperative MRI revealed a characteristic thalamic lesion with a necrotic core (46 mm^3^) and surrounding cytotoxic edema (887 mm^3^). By overlaying the lesion (necrotic core and cytotoxic edema segmented from postoperative day 3 T2-weighted MRI using ITK-SNAP 4.2.2 [20] and normalized to MNI space via ANTs 2.6.0) onto the probabilistic ET map, we found the lesion did not fully encompass the convergence zone of decussating and non-decussating CTT fibers at the thalamic base (Figure 2). This anatomical mismatch likely contributed to both the delayed and incomplete tremor relief. However, significant functional improvement was ultimately achieved. The gradual improvement was unlikely to be related to edema resolution or gliosis but may reflect the time needed for normalization of CTT hyperexcitability and reorganization of the thalamocortical pathway.

Only a few reports have reported outcomes of thalamic lesioning performed after habituation to Vim DBS, and none for PSA DBS. Bahgat et al. [2] reported four patients who experienced symptom progression following initial effective Vim DBS. All but one noted some degree of symptomatic relief after thalamic ablation, although this did not translate into measurable functional improvement. The fourth patient showed neither symptomatic nor functional benefit from thalamotomy. In contrast, Gonzalez et al [3] demonstrated the high efficacy of focused ultrasound (FUS) thalamotomy as a rescue option for failed DBS therapy in two patients. In line with our findings, one patient experienced gradual tremor recurrence 10 years after Vim DBS and underwent unilateral FUS thalamotomy with excellent tremor control at 1-year follow up. The other patient had inefficient DBS (Vim, then PSA) from the outset, which cannot be regarded as habituation. To our knowledge, this is the first report of a successful thalamotomy for PSA DBS habituation, accompanied by delayed postoperative tremor improvement. While our findings primarily address the anatomical and physiological mechanisms underlying DBS failure and thalamotomy efficacy, clinical factors-including disease duration, tremor severity, and individual progression patterns—likely influence treatment outcomes. Also, the single-case design limits broader generalizations.
